# A nomogram model for predicting postoperative prognosis in patients with aneurysmal subarachnoid hemorrhage using preoperative biochemical indices

**DOI:** 10.1186/s12883-024-03774-1

**Published:** 2024-08-03

**Authors:** Zhen Sun, Fei Xue, Kunpeng Wang, Dongbo Zhang, Mengning Dong, Jiandang Zhang

**Affiliations:** 1 The neurosurgery, Nanyang Central Hospital, Nanyang, Henan 473000 China; 2 Shaanxi Provincial People’s Hospital, Xi’an, Shaanxi 710086 China

**Keywords:** Intracranial aneurysm, Subarachnoid hemorrhage, Prognostic nutritional index, Neutrophil/albumin ratio, Prognosis

## Abstract

**Objective:**

The nutritional status and inflammatory responses of patients with aneurysmal subarachnoid hemorrhage (aSAH) play a vital prognostic role. We investigated the relationship between preoperative prognostic nutritional index (PNI)、neutrophil/albumin ratio (NAR)、platelet/albumin ratio (PAR) and other factors and the clinical prognosis of patients who underwent clipping for aSAH and its predictive model.

**Methods:**

The clinical data of 212 patients with aSAH who underwent neurosurgery at Nanyang Central Hospital between 2018 and 2023 were retrospectively analyzed. Based on the Glasgow Outcome Scale (GOS) score at 6 months postoperatively, the patients were categorized into two groups: poor (GOSI-III) and good (GOSIV-V) prognosis groups. Multivariate logistic regression analysis was performed to determine the predictive value of preoperative PNI、NAR、PAR、hyperlipidemia and Glasgow Coma Scale (GCS) for prognosis. Furthermore, nomograms and prognostic prediction models were constructed. Receiver operating characteristic curves and area under the curve (AUC) were utilized to determine the predictive values.

**Results:**

Multivariate logistic regression analysis revealed that PNI (OR = 1.250, 95%CI 1.060 ~ 1.475, *P* = 0.008), NAR (OR = 0.000, 95%CI 0.000 ~ 0.004, *P* = 0.000), PAR(OR = 0.515, 95%CI 0.283 ~ 0.937, *P* = 0.030), hyperlipidemia (OR = 4.627, 95%CI 1.166 ~ 18.367, *P* = 0.029), and GCS(OR = 1.446, 95%CI 1.041 ~ 2.008, *P* = 0.028) are independent risk factors for poor postoperative prognosis. The total score of the nomogram was 200, and the AUC value was 0.972.

**Conclusions:**

PNI and NAR can reflect the nutritional status and inflammatory responses of patients.They are significantly associated with the postoperative prognosis of patients with aSAH. Comprehensively analyzing PNI and NAR combined with other clinical indicators can more effectively guide treatment and help predict prognosis.

## Introduction

Subarachnoid hemorrhage (SAH) accounts for 3% of all strokes [1], with aneurysmal subarachnoid hemorrhage (aSAH) accounting for approximately 85% of spontaneous SAH cases [2]. Particularly in China, where the mortality rate of untreated aSAH patients is 35–40% and the disability rate of aSAH survivors is 50% [3, 4]. Although our understanding of aSAH pathogenesis and ruptured aneurysm treatments, including surgical clipping and endovascular treatment, have improved after decades of research, two treatment methods have the potential toinduce neurological deterioration. aSAH remains a serious threat to global health, 24% of endovascular treatment and 32% of surgical clamping patients still have poor prognosis at 1-year follow-up [5]. Early postoperative brain injury (EBI) and complications associated with delayed brain injury (DBI) are the primary reasons for disability and death among patients with aSAH [6]. The nutritional status and local and systemic inflammatory responses of patients are considered important mechanisms underlying brain injury.

Some studies have revealed that inflammation in the initial stage of aSAH is associated with its pathological process. Neuroinflammation and oxidative damage secondary to aSAH are essential reasons for neurological deficits. The severity of inflammatory reaction is often positively correlated with the prognosis of patients. Blood oozes into the subarachnoid space, releasing large amounts of inflammatory stimuli; these stimuli can activate microglia and trigger an inflammatory cascade [7]. Moreover, the activation of sympathetic pathway and hypothalamus-pituitary-adrenal axis and the release of catecholamines after stroke can promote systemic inflammatory response, resulting in immunosuppression after stroke, resulting in lymphopenia [8]. Furthermore, inflammatory markers, including neutrophil/albumin ratio (NAR), platelet/albumin ratio (PAR), neutrophil/lymphocyte ratio (NLR), platelet/lymphocyte ratio (PLR), and monocyte/lymphocyte ratio (MLR) are associated with aSAH prognosis [9]. In addition, a deterioration in the nutritional status and immune function of patients will affect disease prognosis.

The prognostic nutritional index (PNI) is calculated using serum albumin levels and lymphocyte count and reflects the nutritional and immune statuses. Some studies have revealed that PNI is a reliable prognostic marker for various tumors and other critical diseases; furthermore, it can be utilized to investigate the clinical prognosis of patients with aSAH after clipping [10, 11]. Multiple risk factors affect the prognosis of patients with poorly performing aSAH; however, reliable and direct predictive models to predict the prognosis of these patients undergoing surgical clipping are lacking. Therefore, in this retrospective study, we investigated the preoperative nutritional status and inflammatory responses of patients with aSAH to determine the role of multiple indicators in predicting the prognosis of these patients after clipping. Furthermore, we constructed an early prediction model for poor prognosis to manage patients after aSAH in clinical settings.

## Data and methods

### Patient information and grouping

**Fig. 1:**
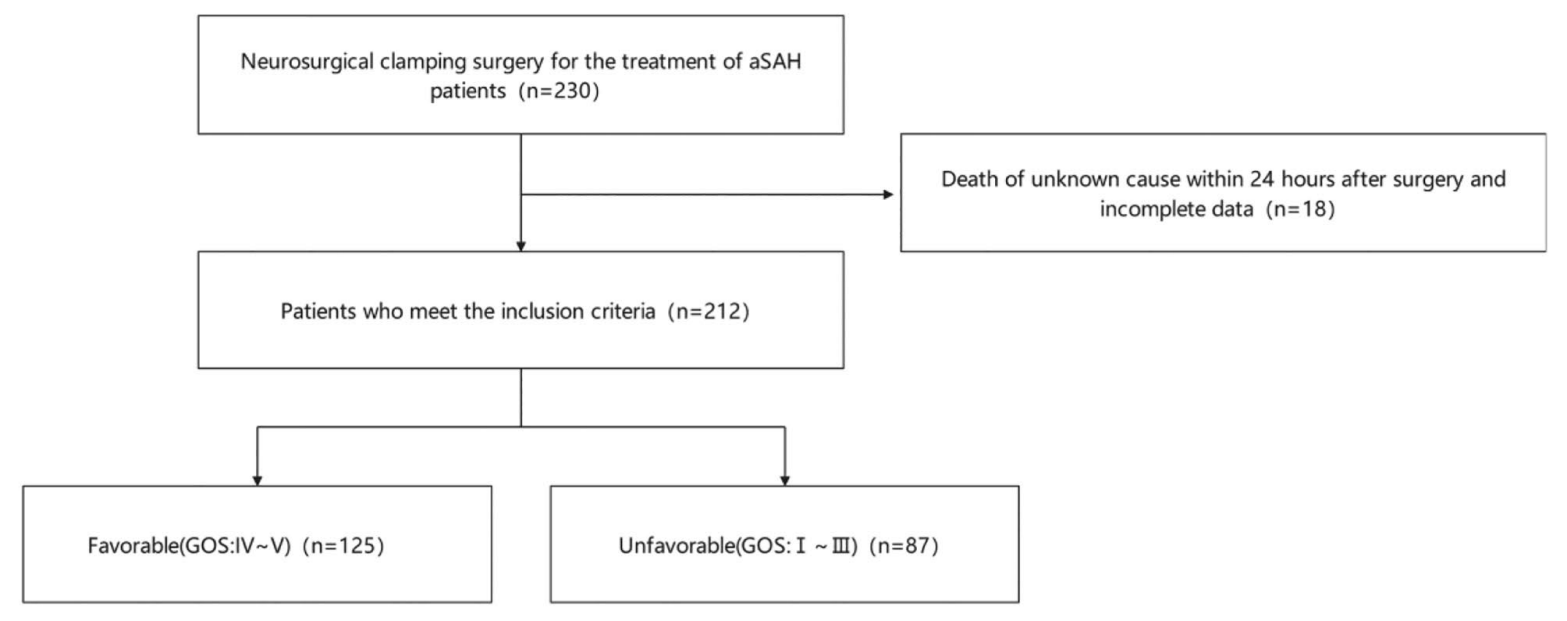
Flow chart for patients included in this study

The clinical data of 212 patients with aSAH (65 men and 147 women) who underwent intracranial aneurysm clipping at the Department of Neurosurgery of Nanyang Central Hospital between January 2018 and August 2023 were reviewed (Fig. 1**)**. The Hunt-Hess grade of the surgical patients was between I-IV grade, and the Glasgow Coma Scale (GCS) score was more than 5. Based on the Glasgow Outcome Scale (GOS) at 6 months postoperatively, the patients were categorized into two groups: poor (GOSI-III) and good (GOSIV-V) prognosis group. This study was conducted according to the principles of the Helsinki Declaration. With the approval of the hospital ethics committee. The informed consent form for surgery was signed by the families of all patients.

The inclusion criteria were as follows: (1) age ≥ 18 years old; (2) improvements in relevant examination parameters after admission and diagnosis of SAH caused by ruptured intracranial aneurysms, as confirmed via computed tomography angiography or digital subtraction angiography; and (3) all patients who underwent intracranial aneurysm clipping within 72 h of onset.

The exclusion criteria were as follows: (1) patients with aSAH because of non-aneurysmal causes such as trauma, arteriovenous malformations, and arteriovenous fistulas; (2) Patients with acute or chronic infections, past autoimmune diseases, previous malignant tumors, uremia, liver cirrhosis, chronic heart disease and chronic lung disease; (3) those receiving anticoagulants or antiplatelet drugs for a long time; and (4) those with recurrent stroke within 6 months of postoperative follow-up; 5. Second operation.

For patients with acute hydrocephalus or intraventricular hematoma(IVH), aneurysmal clipping and contralateral ventricle drainage were performed.The patients with intracranial hypertension were treated with decompressive craniectomy (DC) according to the intraoperative conditions.All patients were transferred to ICU for treatment after operation, and head CT was reviewed within 12 h after operation.

### Data extraction

The clinical data were collected from the hospital information system and follow-up records of Nanyang Central Hospital. Throughout the hospitalization period, all patients who were admitted to the hospital received routine treatment, such as controlling blood pressure, reducing cranial pressure and preventing cerebral vasospasm(CVS), etc., based on the management guidelines for aSAH [12]. Neurosurgical clipping was performed within 72 h after symptom onset. The indications and procedures for the surgery were according to the corresponding neurosurgical clipping guidelines and procedures for intracranial aneurysms [13]. Technologically mature neurosurgeons performed aneurysm clipping using the same surgical specifications and technical standards. Cerebral aneurysm clipping was performed under a microscope. After the surgery, nimodipine was administered to prevent CVS. After discharge, outpatients or telephone follow-up were divided into poor prognosis group (GOSI-III) and good prognosis group (GOSIV-V).

Basic clinical information, including age, sex, smoking history, drinking history, hypertension, diabetes, hyperlipidemia, stroke history, Hunt–Hess grade, IVH, hydrocephalus, postoperative DC, GCS, and aneurysm location, was collected. Owing to limited data, aneurysm location was divided into two types: anterior or posterior circulation.

Neutrophil, platelet, lymphocyte, and monocyte counts and albumin levels were recorded. A preoperative examination was conducted on the first day of admission to measure all blood test indexes. The indexes were determined as follows: PNI = serum albumin (g/L) + 5 × total lymphocyte count (× 10^9^/L), NAR = neutrophils (× 10^9^/L)/albumin (g/L), PAR = platelets (× 10^9^/L)/albumin (g/L), NLR = neutrophils (×10^9^/L)/lymphocytes (× 10^9^/L), PLR = platelets (× 10^9^/L)/lymphocytes (× 10^9^/L), and MLR = monocytes (× 10^9^/L)/lymphocytes (× 10^9^/L).

### Statistical analysis

SPSS25.0 software was utilized to perform statistical analysis. Comparisons between groups with measurements conforming to normal distribution were performed using t-tests, and data are expressed as mean ± standard deviation. On the other hand, the Mann–Whitney U test was performed to compare the non-normal distribution of measurement data between groups; data were expressed as median (M) and interquartile range. Categorical variables were expressed as number of instances and percentages or composition ratios, and comparisons were made using the chi-square test and Fisher’s exact probability test. Indicators that were significant in the univariate analysis were analyzed by multivariate logistic regression analysis. Furthermore, the significant indexes in multivariate analysis were considered independent risk factors for the poor prognosis of patients with aSAH after clipping. The “rms” package of R software was used to construct a column chart to obtain the scores for each indicator. The total score was obtained by adding the scores for each indicator. The higher the total score, the higher the risk of poor prognosis after aSAH. Then, the “pROC” package of R software was used to construct receiving operating characteristic (ROC) curves, and their AUC values were calculated. The larger the area, the stronger the prediction ability. Simultaneously calculate the optimal cutoff value for each indicator to obtain the relatively optimal sensitivity and specificity. The difference was considered statistically significant at a P-value of < 0.05.

When both GCS score and Hunt–Hess grade were included in multiple regression analysis, an offset was observed. The Hunt–Hess grade is recognized as a prognostic indicator, and the GCS score was included in this study. Therefore, only the GCS score was included in the final model.

## Results

### Baseline characteristics

In total, 212 patients with aSAH who underwent aneurysm clipping were included in this study. Among them, 125 (58.96%) had a good prognosis and 87 (41.04%) had a poor prognosis. Age, hypertension, hyperlipidemia, Hunt–Hess grade, IVH, hydrocephalus, postoperative DC, GCS score, aneurysm location, PNI, NAR, PAR, NLR, PLR, and MLR were included in univariate analysis (Table 1).

**Table 1:** Univariate analysis results affecting poor prognosis in 212 patients with aSAH after surgery

Variables	Good outcome(*n* = 125)	Poor outcome (*n* = 87)	Test value	*P*-value
Gender			χ² =0.496	0.289
Male Female	36 89	29 58		
Age (years)	56.32 ± 10.06	60.78 ± 8.87	*t* = 3.333	0.001
Smoke			χ² =0.767	0.243
No Yes	105 20	69 18		
Drink			χ² =1.071	0.203
No Yes	111 14	73 14		
Hypertension			χ² =4.772	0.020
No Yes	62 63	30 57		
Diabetes			χ² =1.159	0.202
No Yes	116 9	77 10		
Hyperlipidemia			χ² =16.448	0.000
No Yes	99 26	46 41		
History of stroke			χ² =0.198	0.411
No Yes	113 12	77 10		
Hunt-Hess	2(2)	3(3)	Z = 7.473	0.000
IVH			χ² =13.930	0.000
No Yes	99 26	48 39		
Hydrocephalus			χ² =28.422	0.000
No Yes	115 10	54 33		
GCS	11(9)	8(5)	Z = 8.512	0.000
DC			χ² =10.549	0.001
No Yes	109 16	60 27		
Location of aneurysm			χ² =9.698	0.002
Anterior circulation Posterior circulation	92 33	46 41		
PNI	48.51 ± 5.47	39.99 ± 5.78	*t* = 10.891	0.000
NAR	0.15 ± 0.07	0.41 ± 0.18	*t* = 12.664	0.000
PAR	5.32 ± 1.79	6.75 ± 2.69	*t* = 4.335	0.000
NLR	5.54 ± 3.81	15.13 ± 8.78	*t* = 9.581	0.000
PLR	172.25 ± 80.87	235.56 ± 106.81	*t* = 4.674	0.000
MLR	0.35 ± 0.22	0.69 ± 0.41	*t* = 6.990	0.000

Multivariate logistic regression analysis revealed that PNI (OR = 1.250, 95% CI: 1.060, 1.475, 0.008), NAR (OR = 0.000, 95% CI: 0.000, 0.004), PAR (OR = 0.515, 95% CI: 0.283, 0.937), hyperlipidemia (OR = 4.627, 95% CI: 1.166, 18.367, 0.029), and GCS score (OR = 1.446, 95% CI 1.041, 2.008) were independent risk factors for poor prognosis postoperatively (Table 2).

**Table 2:** Multivariate logistic regression analysis results affecting postoperative poor prognosis in 212 patients with aSAH

Influencing factors	OR value	95%CI	*P*-value
PNI	1.250	1.060 ~ 1.475	0.008
NAR	0.000	0.000 ~ 0.004	0.000
PAR	0.515	0.283 ~ 0.937	0.030
Hyperlipidemia	4.627	1.166 ~ 18.367	0.029
GCS	1.446	1.041 ~ 2.008	0.028

### Comparison of the ROC curves of different monitoring indexes in predicting poor patient prognosis

**Fig. 2:**
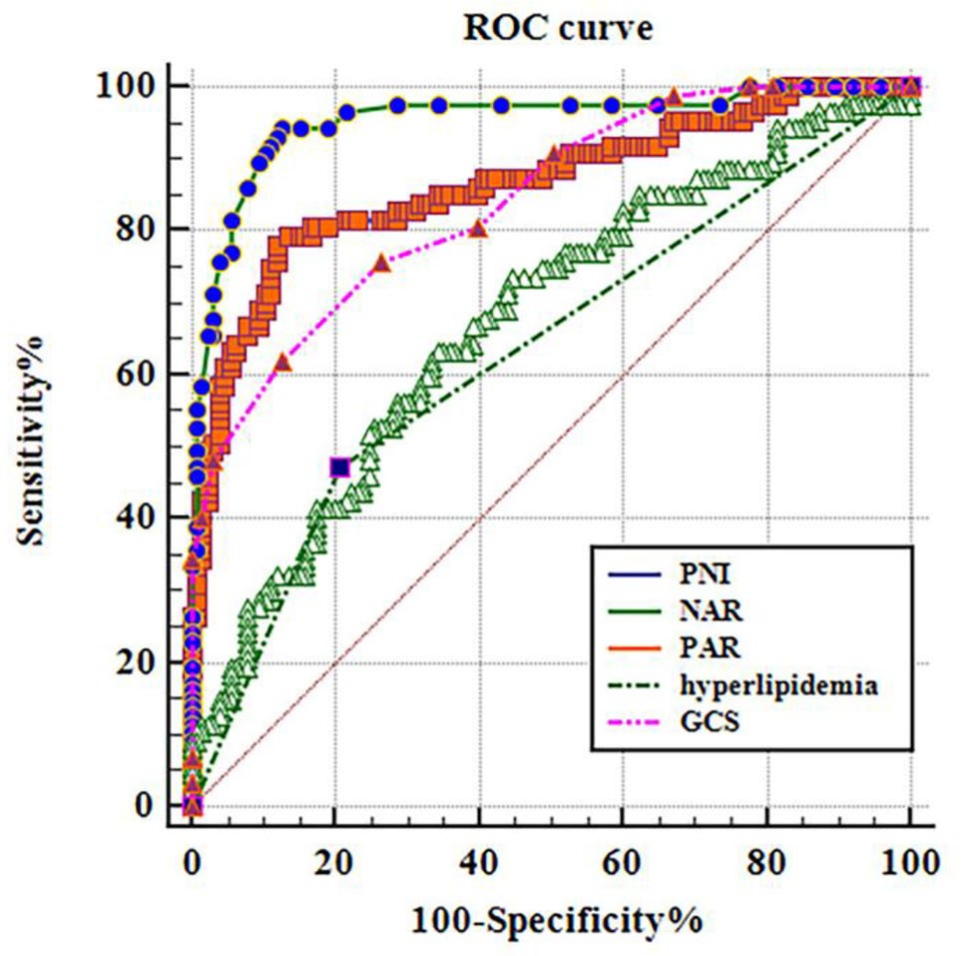
Risk factor index for predicting poor prognosis of postoperative patients with aSAH in the working characteristic curve

The significance of different indexes on the poor prognosis of patients with aSAH postoperatively was analyzed. We observed that PNI (AUC = 0.865, 95% CI: 0.812, 0.908, *P* < 0.001), NAR (AUC = 0.954, 95% CI: 0.916, 0.978, *P* < 0.001), PAR (AUC = 0.675, 95% CI: 0.608, 0.738, *P* < 0.001), hyperlipidemia (AUC = 0.632, 95% CI: 0.563, 0.697, *P* < 0.001), and GCS score (AUC = 0.842, 95% CI: 0.786, 0.888, *P* < 0.001). Therefore, predicting the poor prognosis of patients with aSAH using a combination of several indicators is extremely vital (Table 3; Fig. 2).

**Table 3:** The predictive effect of risk factor indicators on postoperative poor prognosis in aSAH patients

Influencing factors	AUC	cutoff points	sensitivity(%)	specificity(%)	95%CI	*P*-value
PNI	0.865	43	78.16	88.00	0.812 ~ 0.908	<0.001
NAR	0.954	0.21	94.25	87.20	0.916 ~ 0.978	<0.001
PAR	0.675	5.6	63.22	65.60	0.608 ~ 0.738	<0.001
Hyperlipidemia	0.632	-	47.13	79.20	0.563 ~ 0.697	<0.001
GCS	0.842	9	75.86	73.60	0.786 ~ 0.888	<0.001
All indicators are combined	0.972	-	92.00	94.25	0.940 ~ 0.990	<0.001

### Risk assessment model for the poor prognosis of patients with aSAH after clipping

**Fig. 3:**
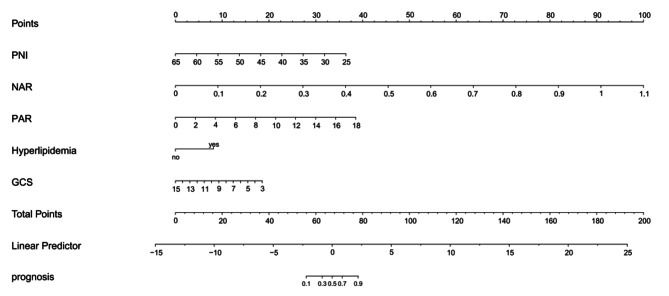
A nomogram based on multiple logistic regression model for predicting poor prognosis in aSAH neurosurgical clamp patients

The significant indicators in logistic regression and ROC curve analyses were included in a nomogram to construct a prediction model for disease risk (Fig. 3). The total score of the nomogram was 200. When the total score was > 78, the risk of poor prognosis of patients with aSAH after clipping was > 90%. The AUC value of the nomogram was 0.972 (95% CI: 0.940, 0.990, *P* < 0.001). This value is significantly higher than that of other single-index models. This suggests that the constructed prediction model exhibits good accuracy and clinical application value.

## Discussion

aSAH is a common cerebrovascular disease requiring neurosurgery that threatens human health [14]. The management of EBI and DBI-induced postoperative complications, including CVS, delayed cerebral ischemia (DCI), Acute hydrocephalus, secondary brain swelling, and aneurysm rerupture bleeding, remains challenging [15, 16]. Multiple risk factors influence the prognosis of patients with aSAH. However, there is a lack of reliable and direct predictive models to predict the prognosis of aSAH patients undergoing surgical clamping. Therefore, it is important to focus on the prognostic outcomes of aSAH patients to improve their quality of life and survival. The aim of this retrospective observational study was to investigate the relationship between potential clinical risk factors and prognosis after aSAH clamping. Multivariate logistic regression and ROC curve analyses revealed that preoperative PNI, NAR, RAR, hyperlipidemia, and GCS score are important factors affecting the prognosis of patients with aSAH at 6 months postoperatively. Using these five risk factors, we constructed a nomogram to predict the risk of poor prognosis, facilitating the decision-making processes for management and treatment in clinical settings. The nomogram was found to be well calibrated, predictive and clinically applicable through internal validation. Therefore, our predictive model can help predict adverse outcomes and develop the best treatment strategy for patients with aSAH with poor prognosis after aneurysm clipping.

As a clinical prognostic indicator for patients, PNI is primarily used for various tumors and in major surgical research fields; however, studies on acute cerebrovascular diseases primarily focus on ischemic stroke [17]. In our study, we observed that preoperative PNI is a valuable prognostic predictor for patients with aSAH at 6 months after clipping and that high PNI functions as a protective factor (*P* < 0.05). PNI integrates serum albumin levels and lymphocyte count to reflect the immune responses and nutritional status of patients [18]. Serum albumin is a commonly used nutritional index, and several studies have revealed that it serves as a predictive index for the prognosis of patients with stroke. Serum albumin can increase plasma osmotic pressure, decrease brain edema, maintain blood–brain barrier (BBB) integrity, and improve the internal environment [19, 20]. Lymphocytes are essential leukocyte subsets that participate in host defense and adaptive immunity. Studies have revealed that a low lymphocyte count is a predictor of poor prognosis. Unlike neutrophils that promote inflammation, lymphocytes can inhibit inflammation [21]. As an indicator of cellular immunity, a low lymphocyte count suggests a decreased immune level. However, compared with serum albumin or lymphocytes alone, PNI is a better indicator of a patient’s nutritional and immune status than serum albumin or lymphocytes alone. A low preoperative PNI reflects a decrease in serum albumin or lymphocyte counts, demonstrates a severe inflammatory response and immunosuppression, and indicates poor general condition and reduced nutrition and immunity [22, 23]. Based on our findings, a preoperative PNI value of < 43 significantly increases the risk of adverse outcomes in patients with aSAH undergoing neurosurgical clipping.

Inflammatory cytokines reached the peak within 48 h after aSAH onset. Excessive inflammatory reactions lead to the poor prognosis of patients with aSAH [24]. In the present study, we analyzed many inflammatory indicators. Among them, NAR and PAR are significant. Elevated neutrophil counts are associated with poor prognosis and in-hospital complications of aSAH [25]. Neutrophil products such as free radicals and proteolytic enzymes mediate BBB damage. Furthermore, the release of extracellular traps by neutrophils exacerbates inflammation, destroying the BBB and aggravating damage to surrounding neurons and other brain cells [26]. Increased neutrophil counts are associated with the poor prognosis of patients with aSAH, whereas hypoproteinemia is associated with infection during hospitalization. This may be the mechanism by which NAR predicts aSAH prognosis [9].

Platelets are vital factors in the coagulation system. In addition to hemostasis and coagulation, platelets also participate in inflammation and atherosclerosis development [27]. The higher the PAR level, the higher the platelet count; this reflects the high inflammatory response state and susceptibility to thrombosis [28]. Increased the risk of DCI, resulting in a poor prognosis. Furthermore, a high PAR level indicates a low albumin count, suggesting malnutrition and high inflammation and predicting poor clinical outcomes [29].

High lipid levels increase the risk of poor aSAH prognosis. Vascular smooth muscle cells can repair the damaged aneurysm wall and delay inflammation progression. Presumably, high lipid levels promote inflammation of the aneurysm wall and lead to vascular smooth muscle cell death [30].

GCS score is a simple and effective tool to evaluate the consciousness level of patients; it can help standardize the clinical evaluation of patients with stroke and facilitate patient management. The prognosis of patients with aSAH significantly correlates with GCS score [31]. In the present study, we observed that GCS scores can effectively predict the poor prognosis of patients with aSAH after microsurgical clipping. Compared with other scoring systems, preoperative GCS can better reflect the consciousness level of patients. The GCS score should be closely monitored to reflect the severity of the patient’s condition and appropriate treatment measures should be formulated to improve the prognosis. In dynamic diseases such as aSAH, continuous GCS score monitoring may be more clinically relevant than assessing the Hunt–Hess grade at admission [32].

Neutrophils, albumin, platelets and hyperlipidemia are all indicators that are easy to intervene in clinic.In clinic, anti-inflammatory treatment methods such as Non-steroidal anti-inflammatory drugs (NSAIDs) and glucocorticoid can be selected according to the condition of patients.NSAIDs can effectively reduce vasospasm and neurological dysfunction.Glucocorticoids may help to reduce the production of vasospasm and IL-6, and promote a good prognosis of patients undergoing microsurgical clipping [33, 34]. Patients with hypoalbuminemia were treated with albumin injection, 1.25 g / kg / day. Furthermore, studies have demonstrated that albumin regulates its neuroprotective effect by decreasing brain damage and improving neurovascular remodeling. In addition, it can inhibit inflammation, scavenge free radicals, and produce free radicals, Improve the prognosis of patients [35, 36]. For patients with high platelet level, antiplatelet therapy should be given as early as possible according to clinical conditions to reduce the formation of microthrombosis, reduce the risk of DCI, and improve the prognosis [37]. Patients with hyperlipidemia can be treated with statins, Studies have revealed that statins can inhibit leukocyte migration and proliferation into blood vessels, activate cytokines, upregulate endothelial nitric oxide synthase expression and activity, improve endothelial reactivity, increase cerebral blood flow, and play an antioxidant role. Furthermore, a short statin course (lasting 2 weeks) may improve neurological prognosis. Statins are associated with decreased CVS. Although statins can increase bacteremia, based on the pathophysiological characteristics and prognosis of CVS, 2 weeks may be the best time window for statins to treat aSAH [38].

Our study has some limitations. This was a single-center retrospective cohort study with a small sample size and possibly biased results. Furthermore, we conducted patient follow-up only for 6 months. When establishing predictive models for aSAH, more attention should be paid to the nutritional status and inflammatory indicators of patients. In this study, the neutrophil count, platelet count, lymphocyte count, and albumin level were routine in-patient examinations for patients with aSAH. The calculation methods are simple, convenient to obtain, and inexpensive. Furthermore, it is easier to perform clinical interventions on the indicators and convenient to use in hospitals at all levels. In addition, we preliminarily demonstrated that the combined prediction of PNI and NAR improves the predictive performance of existing predictive models and deserves further clinical validation in additional prospective multicenter studies.

## Conclusions

Using univariate and multivariate logistic regression analyses, we identified five clinical predictors, namely, PNI, NAR, PAR, hyperlipidemia, and GCS score, as well as several risk factors affecting the prognosis of patients with severe aSAH and the interactions among these factors. Furthermore, we developed a nomogram using these five predictors. Internal validation revealed the good accuracy and clinical utility of the model in helping clinicians assess the prognosis of patients with aSAH undergoing surgical clipping.

## Data Availability

Data is provided within the manuscript or supplementary information files.
